# Therapeutic drug monitoring for valproate: deriving a novel formula for calculation of free concentration

**DOI:** 10.1007/s00228-024-03741-2

**Published:** 2024-08-16

**Authors:** Erna Pretorius, Paulina van Zyl, Gina Joubert

**Affiliations:** 1 Department of Pharmacology, Faculty of Health Sciences, University of the Free State, Bloemfontein, South Africa; 2 Department of Biostatistics, Faculty of Health Sciences, University of the Free State, Bloemfontein, South Africa

**Keywords:** Calculation, Formula, Free concentration, Therapeutic drug monitoring, Total concentration, Valproate

## Abstract

**Background:**

Monitoring free valproate concentrations, as with other highly protein-bound anticonvulsants, is essential in clinical situations where protein binding may be disrupted. Conversion of measured total concentrations to approximate free concentrations offers a cost-effective alternative. This study evaluated the relationship between total and free valproate concentrations for discordance and the impact of key determinants. A novel formula was devised that incorporates significant variables.

**Methods:**

A multicentre, cross-sectional observational analytical study included 101 subjects 18 years and older using valproate for 6 months or longer. Participants were recruited from private and public sector healthcare settings from primary to tertiary level in, South Africa, during 2017–2019.

**Results:**

Free valproate concentrations could be measured for 84 subjects. Discordance for concomitant total and free valproate concentrations was 79.1%. Among 19 participants with elevated free concentrations, 15 (78.9%) had total valproate concentrations within the recommended reference range. Calculations based on the study-derived formula were more accurate in predicting free valproate concentration than previously proposed methods.

**Conclusion:**

This study proposes that the novel formula for calculating free valproate enables more accurate prediction.

## Introduction

Valproate (VPA) is widely utilized for its broad therapeutic applications and reasonable safety profile when prescribed as the first-line treatment for epilepsy and as a mood stabilizer [1]. Presently, therapeutic drug monitoring (TDM) for VPA primarily focuses on measuring the total VPA serum concentration, encompassing both the inactive protein-bound component and the active free concentration with the free fraction [2] expressed as a percentage of the total and free concentration [3].

Several methods for directly measuring free VPA concentration have been described [4], but their adoption remains limited. While the routine measurement of free drug concentration may be desirable in the clinical setting, it has not been achieved in routine laboratory practice. This is due to cost, availability of testing facilities, and expertise. Transportation and storage requirements for blood samples may also pose logistical challenges in settings with limited infrastructure and due to prioritization of resources where competing demands for healthcare resources and free VPA level testing may not be prioritized compared to other healthcare needs. On a global scale, funding research to minimize drug adversities is often considered less advantageous and less profitable [5].

As an alternative, several authors have proposed formulae for estimating free VPA concentration [3, 6–8]. However, these estimations have limitations, particularly with method-specific factors, such as those related to albumin measurements. Furthermore, the presence of other highly protein-bound drugs, such as aspirin, nonsteroid anti-inflammatory drugs (NSAIDs), and warfarin, can displace VPA from protein binding sites, potentially affecting the accuracy of estimated free VPA levels [7]. Moreover, factors such as elevated free fatty acids, aging, hypoalbuminemia, drug interactions, uremia, hyperammonaemia [9], pH changes, and lipid presence can further complicate the estimation of free VPA levels. These complexities underscore the challenges in accurately estimating free VPA levels using formulae, as they may not fully account for all variables that affect protein binding dynamics in clinical settings.

Monitoring the total VPA concentration has traditionally therefore been the cornerstone of VPA TDM. This approach is grounded in the expectation that total VPA levels increase in a linear fashion with dosage increments [10]. Additionally, valproate exhibits concentration-dependent plasma protein binding, which typically results in a linear increase in total VPA concentrations proportional to daily dosage. However, this relationship appears to reach saturation at a distinct, inter-individually determined physiological point [4]. Stable protein binding is reported within total VPA concentrations ranging from 20 to 60 μg/mL, with an approximate free fraction of 10%. Beyond this range, there is a notable non-linear progressive increase in total VPA concentrations [3].

Conversely, the relationship between free VPA concentration and total VPA concentration appears to be considerably more intricate as demonstrated by Drisaldi et al. [11], with total VPA being only 56.9% concordant with the concomitant free VPA categories. Riker et al. [9] described an extremely high variation in free fraction monitoring (ranging from 15 to 89%) in 15 intensive care unit (ICU) patients receiving VPA with total VPA reflecting below the recommended reference range (RRR). In addition, the RRR of total VPA concentration of 50–100 μg/mL [12, 13] was derived from small studies [4] that did not consider the role and measurement of free VPA concentration. The discordance between total and free VPA levels is particularly concerning in vulnerable populations such as patients in ICUs and the elderly where TDM is imperative. Clinical consequences of this discordance include adversities and misinterpretation in the clinical decision-making process if only the total VPA is monitored. Consequently, personalized medicine approaches have become crucial in tailoring treatment strategies based on individual patient characteristics, underscoring the importance of monitoring free VPA concentrations in addition to total levels [14, 15].

The primary objective of this study was to measure total and free VPA concentrations and calculate VPA free fraction in adults on VPA for a minimum of 6 months. A secondary goal was to examine the variability in these measurements among patient groups and to investigate factors influencing these outcomes. Ultimately, the study aimed to devise a novel approach for estimating free VPA concentration using total VPA levels and key variables that significantly impact free VPA concentration.

## Methods

A cross-sectional observational and analytical study design was used. Approval was obtained from the Health Sciences Research Ethics Committee (HSREC) of the University of the Free State in Bloemfontein, South Africa (ethics approval reference number [removed to ensure blind review]). Written informed consent was obtained from all participants.

The population consisted of adult patients on VPA or its derivatives for a minimum period of 6 months, using VPA for one of the following chronic conditions: epilepsy, bipolar mood disorder, migraine, hyperkinetic movement disorders or neuropathic pain and peripheral neuropathy. Exclusion criteria were concomitant oral anticoagulation agents, treatment with additional antiepileptic drugs, minors younger than 18 years of age, pregnancy, current treatment with carbapenem antibiotics, and known liver function impairment. As the study did not have only one main outcome of interest, a sample size of 100 or more was considered to provide sufficiently narrow confidence intervals for the diversity of prevalence levels and adequate numbers for subgroup analyses. Participants were recruited from multiple centers including private and public sector healthcare settings from primary to tertiary level in Bloemfontein, South Africa, during the 3-year period 2017–2019.

The demographic data collected comprised sex, age, and ethnicity. Daily VPA dose, indication for VPA use, comorbidities, and concomitant medication and alcohol use were also recorded. Blood samples were collected to determine total and free VPA concentrations, as well as albumin concentration.

Total VPA concentrations were determined using centrifugation in a fixed-angle rotor (Statspin Express 3 Centrifuge, Beckman Coulter; Brea, CA, USA). Total VPA concentration in the supernatant was then measured with the Beckman Coulter AU680 Clinical Chemistry Analyser immunoassay (Beckman Coulter; Brea, CA, USA) homogeneous enzymatic immunoassay. For free VPA concentration, the supernatant sample of 500 µL serum was transferred to VivaSpin500 30-kDa centrifugal concentrator tubes (Fisher Scientific; Waltham, MA, USA) and centrifuged in a fixed-angle ultracentrifuge rotor (Heraeus Pico 17, Thermo Scientific; Waltham, MA, USA) separating the free VPA from the albumin-bound VPA molecules. The ultrafiltrate was subsequently analyzed for free VPA using the Beckman Coulter VPA immunoassay. The precision of the assay was reported as 2.7 to 4.0% at low concentrations of 28.0 and 12.4 µg/mL, 2.9% at a concentration of 77.9 µg/L, and 2.7% at a concentration of 124.0 µg/mL on quality control material. The instrument and assay were performed within expected limits on the external quality control peer-data programs (RIQAS) as proof of accuracy.

Albumin concentrations were determined in the ultrafiltrate by means of a nephelometric assay on an Immage® 800 Protein Chemistry Analyser (Beckman Coulter; Brea, CA, USA) with a lower limit of detection of 2 µg/mL. Albumin concentrations below the assay’s limit of detection were accepted as successful ultracentrifugation. Filter failure was observed in seven of the samples during ultracentrifugation. For these seven samples, the free VPA predicted value using the respective formulae proposed by Nasreddine et al. [3], Doré et al. [6], and Parent et al. [16] was used.

The results of total VPA and free VPA determinations were divided into three categories, for total VPA: below the RRR (less than 50 µg/mL), within the RRR (50–100 µg/mL), and above the RRR (above 100 µg/mL). For free VPA results, similar categories were defined as below the RRR (less than 4 µg/mL), within the RRR (4–12 µg/mL), and above the RRR/elevated (above 12 µg/mL). A discordance was defined as any mismatch in the concomitant categories between the measured and estimated values.

Numerical data were summarized by medians, ranges, and interquartile ranges (IQRs) due to skew distributions. Categorical variables were summarized by frequencies and percentages. Subgroup comparisons of numerical variables were done using the Mann-Whitney or Kruskal-Wallis nonparametric tests. Spearman’s rank correlations were calculated for the association between numerical variables. Subgroup comparisons of categorical variables were done using chi-square or Fisher’s exact tests. Linear and polynomial regression were performed to predict free VPA. Kappa values with 95% confidence intervals (CIs) were estimated to assess the agreement between the categorization of free VPA values according to different formulae. A *p*-value of < 0.05 was considered statistically significant.

## Results

The data of 101 participants were included for analysis. The demographic data are presented in Table 1. Of the original 101 blood samples, free VPA concentration could be accurately determined for 84 and total VPA concentration for 101 samples (Table 2). For six samples, the values were first calculated/estimated as the method for free VPA measurements had been introduced later in the study. Seven samples were excluded due to filter failure during ultracentrifugation, and for four samples, it was indicated that the value was < 3.0 µg/mL, which is the lower detection limit. These latter four cases were included when free VPA concentrations were categorized as below/normal the RRR.

**Table 1:** Summary of demographic and clinical variables (*n* = 101)

Variables	Median (IQR)
Age (years)	48 (35–62)
Daily prescribed VPA dose (mg) (*n* = 98)	1 000 (600–1000)
	Frequency (%)
Sex	
Female	56 (55.4)
Male	45 (44.6)
Ethnicity	
Black	45 (44.6)
White	46 (45.5)
Mixed race	10 (9.9)
Indication for use (*n* = 99)	
Epilepsy	68 (68.7)
Migraine	21 (21.2)
Bipolar mood disorder	14 (14.1)
Pain and other	7 (7.0)
Comorbidities	
Hypertension	33 (32.7)
Anxiety	24 (23.8)
Hypoalbuminemia	10 (9.9)
Valproate (VPA) monotherapy	24 (23.8)
Concomitant medication	
Highly protein-bound medication	61 (62.2)
Concomitant medication not affecting	36 (35.6)
Concomitant medication affecting CYP450	38 (37.6)
On VPA and other medication uncertain	3 (3.0)

*IQR* interquartile range

**Table 2:** Summary of valproic acid (VPA) measurements

Variable	Range	Median	IQR	RRR/therapeutic range
Total VPA (µg/mL) (*n* = 101)	5.0–125	54.0	34–74	50–100
Free VPA (measured) (µg/mL) (*n* = 84)	3.1–22.6	7.5	5.1–11.6	4.0–12.0*
VPA free fraction (%) (*n* = 84)	8.4–34.5	13.9	12.1–17.4	–

*IQR* interquartile range, *RRR* recommended reference range

^*^Wallenburg et al.[17]

**Fig. 1:**
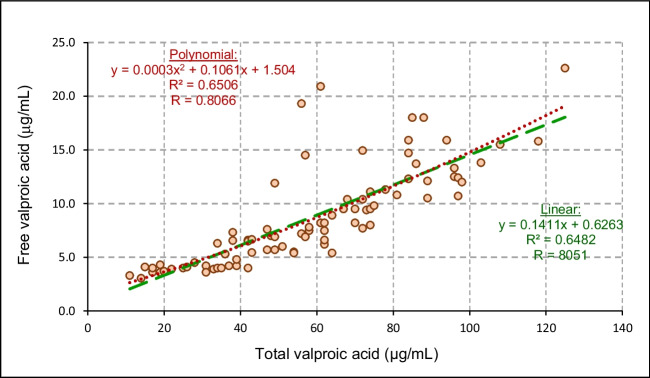
Relationship between free valproic acid and total valproic acid concentrations (*n* = 84). The red trendline presents the polynomial correlation, and the green line presents the linear correlation

The statistical relationship depicted in Fig. 1 indicates that there was no significant difference between the polynomial (dotted line) and linear (solid line) correlations of free VPA versus total VPA. The *R*^2^ and *R* values were similar for both the dotted and solid lines.

For 12 (25.5%) of the 47 total VPA samples below 50 µg/mL, the free VPA was either lower than the detection limit of < 3.0 µg/mL or had to be estimated. Consequently, 35 free VPA measurements were available to compare to their concomitant VPA concentrations. Free VPA concentrations within the RRR were present in 27 of these 35 participants indicating a discordance of 77.1%. Furthermore, of the 19 (*n* = 19/84; 22.6%) participants with elevated free VPA concentrations, 15 (78.9%) had total VPA concentrations within the RRR, indicating a discordance of 78.9% (*n* = 15/19) for the total VPA concurrent categories.

**Fig. 2:**
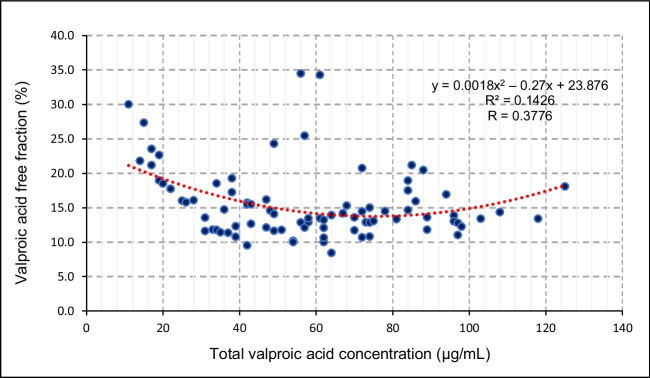
Scatter plot of the relationship between valproic acid free fraction (%) plotted against total valproic acid concentration (µg/mL). The red trendline represents a polynomial correlation

Figure 2 demonstrates the statistical, non-linear relationship between the free fraction and total VPA in the sample population. The free fraction was estimated per category of total VPA concentration indicating that the median free fraction was the highest (22.6%) in the ultra-low total VPA category (< 20 µg/mL), with a median of 13.3% as depicted by Table 3. The difference in free fraction between the total VPA categories was statistically significant (*p* = 0.0011). Furthermore, the free fraction was analyzed according to unique demographic variables. Table 4 demonstrates that progression in age was positively associated with an increase in the median percentage of the free fraction. The median free fraction was increased in the White group compared to the other ethnic groups as shown in Table 4. This difference was statistically significant (*p* = 0.0410).

**Table 3:** Free fraction (%) of detectable free valproic acid concentration per category of total valproic acid concentration and sex (*n* = 84)

	Range (%)	Median free fraction (%)	IQR (%)
Total VPA concentration			
Ultra-low: < 20 µg/mL (*n* = 7)	18.9–30.0	22.6	21.2–27.3
Low: 20–50 µg/mL (*n* = 28)	9.5–24.3	14.6	11.8–16.1
Normal: 50–100 µg/mL (*n* = 45)	8.4–34.5	13.3	12.1–15.0
High: > 100 µg/mL (*n* = 4)	13.4–18.1	13.9	13.4–16.2
Sex			
Female (*n* = 49)	9.5–30.0	14.4	12.2–16.2
Male (*n* = 35)	8.4–34.5	13.5	11.8–19.3

IQR, interquartile range.

**Table 4:** Valproic acid free fraction according to age group (*n* = 84) and ethnic groups

	Number of participants	Median VPA free fraction (%)	IQR (%)
	*n* (%)		
*X*-axis bin range (age in years)			
< 20	1 (1.2)	11.6	–
20–30	16 (19.0)	12.5	11.7–13.4
31–40	14 (16.7)	13.2	11.8–14.9
41–50	15 (17.9)	14.2	12.5–17.1
51–60	16 (19.0)	14.0	12.9–17.7
61–70	15 (17.9)	17.0	14.0–19.6
71–80	5 (5.9)	15.5	13.9–20.4
>80	2 (2.4)	23.0	19.1–26.4
Ethnicity			
African Black	38 (45.2)	13.4	12.1–14.6
Mixed race	6 (7.1)	13.2	12.1–14.5
White	40 (47.6)	15.9	12.8–19.9

*IQR* interquartile range

Co-medication was recorded in 98 individuals as shown in Table 1, and 62.2% (*n* = 61) were on highly protein-bound medication. The median prescribed daily dose of VPA was 1000 mg (IQR 600–1000 mg) with a range of 100–2000 mg (Table 1).

**Fig. 3:**
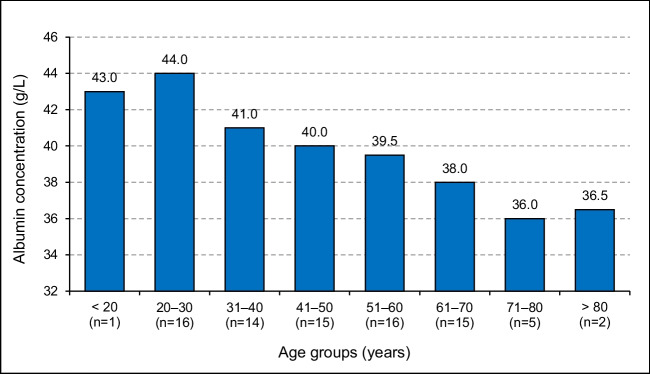
Median albumin (g/L) concentration plotted against age grouped in 10-year intervals (*n* = 84)

Hypoalbuminemia was observed in 10 (9.9%) of the 101 participants. For one of these patients, the free VPA could not be measured. For five of these hypoalbuminemic individuals, the total VPA concentrations were within the RRR, while the free VPA was elevated. For the other five participants, the total VPA concentration was below the RRR, while their free VPA was within the RRR. Albumin concentration decreased with aging, with the lowest medians observed among participants ≥ 60 years of age (Fig. 3).

The daily prescribed dose was correlated to the free VPA concentration with the Spearman rank correlation indicating a moderate positive correlation, with *r* = 0.39 and *p* = 0.0002. Participants with elevated free VPA concentrations presented with statistically significantly (*p* = 0.0056) overall higher values for the daily prescribed VPA dose (median 1000 mg; IQR 1000–1400 mg), compared to those with free VPA concentrations within the RRR (median 800 mg; IQR 600–1000 mg). The Spearman rank correlation showed a weak positive relationship between age and free VPA concentration, with *r* = 0.13 (*p* = 0.2101). A higher percentage of male participants (31.6%) presented with elevated free VPA concentration compared to females (14.0%), although this difference did not reach statistical significance (*p* = 0.0669). Ethnicity did not demonstrate any significant difference regarding elevated free VPA concentration (*p* = 0.4841). The free VPA concentration was elevated in 20.0% of the black participants, 14.3% of the mixed race, and 24.4% of the White participants.

The Parent [16], Nasreddine [3], and Doré [6] formulae for the estimated values were compared to the measured free VPA concentration. The agreement between the categorization according to the Parent formula for estimated free VPA and measured free VPA was poor (kappa [κ] = 0.20; 95% CI 0.09; 0.31), and moderate for the Nasreddine formula [3] (κ = 0.54; 95% CI 0.40; 0.67) and the Doré formula [6] (κ = 0.48; 95% CI 0.35; 0.61).

The study-devised formula was calculated as

$$Free\;VPA=11.227+0.140\times total VPA(\mu g/mL)+0.006\times age (years)-0.274\times albumin (g/L),$$

where total VPA represents the total VPA concentration.

**Fig. 4:**
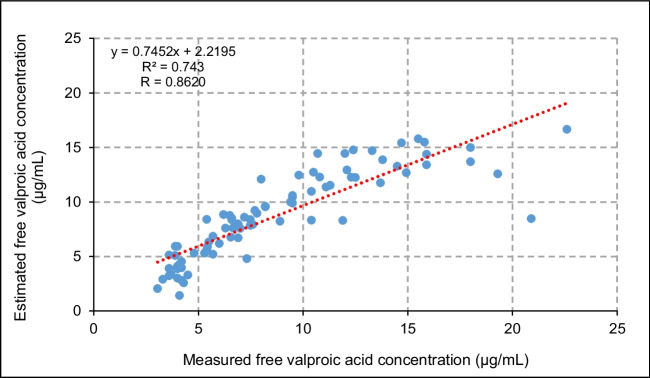
Scatter plot of the relationship between the estimated free valproic acid concentration using the new study-derived formula, compared to the measured free valproic acid concentration. The dotted red trendline presents the data according to a linear equation

This formula led to *R*^2^ = 0.74 and *R* = 0.86. The predicted median free VPA concentration was 8.6 μg/mL (IQR 5.9–11.0 µg/mL) compared to the measured median value of 7.5 μg/mL (IQR 5.1–11.6 µg/mL), indicating an overestimation of the free VPA. The difference ranged from − 11.8 to 4.1, with a median of 0.64 µg/mL and IQR of − 0.52–1.4 µg/mL. Figure 4 shows the predicted values against the measured values. The agreement between the categorization of estimated free VPA and measured free VPA was moderate (κ = 0.64; 95% CI 0.49; 0.78).

## Discussion

The present study underscores a significant discordance between measured total and free VPA concentrations, particularly in relation to the RRR for free VPA as proposed by Wallenburg et al. [17], which was consistent with findings reported in the literature. Specifically, the study found that relying solely on total VPA concentration correctly predicted a concurrent free VPA concentration below the RRR in only 22.9% (8 out of 35) of participants. Conversely, 79.1% (27 out of 35) of participants would be considered within the target RRR when, actually, the bioactive-free VPA was above the RRR when only total VPA concentrations were monitored. This suggests that when TDM is needed, relying only on total VPA concentrations could lead to a misinterpretation of values being within the target RRR range for these individuals.

In particular, this discrepancy becomes clinically significant in scenarios where TDM is crucial. Notably, for vulnerable populations in the present study, which included the elderly and those taking multiple medications, especially highly protein-bound co-medications, the findings regarding discordance related with those reported by Wallenburg et al. [17] and Webb et al. [14], underscoring the limitations of exclusively monitoring total VPA concentration. For those populations where TDM is mandatory, relying solely on total VPA measurements may not suffice and may lack in providing a complete picture, which could lead to inappropriate dose adjustments and suboptimal treatment outcomes. It is crucial to consider the clinical impact of the free, biologically active compound of VPA to ensure optimal treatment outcomes as elevated free VPA concentrations can propense clinical adversity [17]. In a recent large multicentre retrospective cohort study, Webb et al. [14] demonstrated an independent association between free VPA concentrations and clinical adversities in critically ill patients. These adversities included thrombocytopenia and hepatotoxicity. Importantly, their study adjusted for variables that affect protein binding dissociation, indicating that the free VPA concentration itself was a significant factor in predicting these adverse outcomes.

Furthermore, the present study explored the relationship between free and total VPA concentrations, aiming to identify predictable parameters for discordance.

The present study observed increased free VPA fractions occurring randomly and unpredictably within both the total and free VPA RRR. Notably, when grouping according to the concomitant total VPA concentration, the median free fraction was the highest for the ultra-low total VPA concentration group at 22.6% versus 13.3% for those within the RRR for total VPA (*p* = 0.0011).

A possible explanation for the difference between the findings for the present study demonstrating the non-linear and at times erratic relationship between total VPA and free fraction, and the findings reported by Nasreddine et al. [3], could be that in their study, they did not measure albumin concentrations. This was noted as a major limitation, precluding the extrapolation of their results to patients with abnormal serum albumin concentrations. Furthermore, for their study, VPA was prescribed as monotherapy, they did not indicate whether their participants had been on any concomitant medication, which could have altered the VPA kinetics, including drug displacements and/or drug metabolism. They also excluded patients with chronic medical conditions [3]. These exclusions could explain the difference in the results, as in the present study, chronic conditions such as tuberculosis and diabetes mellitus, among others, were included. Medications such as antiretroviral therapy and tuberculosis regimes were also included, and alcohol use was not an exclusion criterion for the present study. In the present study, 62.2% (*n* = 61) of patients were using highly protein-bound co-medications. VPA was also used as monotherapy in the present study, as the use of other AEDs was an exclusion criterion. These complexities highlight the need for nuanced clinical approaches in assessing VPA’s therapeutic effects, especially considering altered pharmacokinetics beyond protein binding saturation.

Furthermore, as emphasized by Lampon and Tutor [2], an increase in free VPA concentration does not necessarily lead to increased drug clearance per unit time and a subsequent decrease in total VPA concentration. The fate of the unbound state depends on individual pharmacokinetic factors at that moment, including renal clearance (affected by renal failure and dialysis), albumin concentration, protein binding, and drug metabolism (influenced by liver disease). These factors can either increase or maintain free VPA concentration, potentially resulting in a decreased total VPA concentration. The pharmacological effect may be similar to increasing total VPA concentration in patients with normal albumin levels [2, 18]. In the present study, a moderate dose-dependent positive association was observed between free VPA concentration and the daily prescribed VPA dose (Spearman’s correlation coefficient *r* = 0.39, *p* = 0.0002), whereas the free fraction did not correspond with the VPA dose (*r* = 0.5, *p* = 0.6570). These findings were similar to those reported by VandenBerg et al. [19], who also found that the VPA dose did not correlate with the VPA free fraction. There was, however, a significant difference (*p* = 0.0410) between the different ethnic groups in the present study, with a higher median VPA free fraction of 15.9% present in White participants versus 13.2% in mixed-race participants and 13.4% in African-Black participants. This is the first study to evaluate the relationship between ethnicity, the total VPA concentration, free VPA concentration, and VPA free fraction. The research emphasizes the complexities in interpreting VPA concentrations solely based on total levels. These findings advocate for incorporating free fraction measurements, considering patient demographics such as age and ethnicity, into clinical practice for more accurate TDM and dosing strategies. The findings also highlight the necessity of further evaluation regarding the importance of ethnicity being considered when estimating free VPA.

In this study, univariate linear regression analyses revealed that total VPA concentration, serum albumin levels, and patient age were the primary determinants affecting the free fraction of VPA. These findings were consistent with those of Doré et al. [6]. The study introduced a novel formula for estimating free VPA concentrations, integrating both age and albumin concentration. This new method improved the accuracy of free VPA estimations.

Moreover, the sample size of the study was more representative being larger compared to that of Doré et al. [6] (101 versus 41 participants, respectively), which was the second most accurate in terms of the discordance for the measured and estimated free VPA (κ = 0.48; 95% CI 0.35; 0.61). The Parent formula [16] showed a better correlation compared to the findings published by Riker et al. [9], who reported an underestimation of the free fraction with a bias of − 11.9 mg/L using the Parent formula. However, the Parent formula [16] is considerably limited as it assumes normal serum albumin concentrations in the absence of renal failure, jaundice, and high total VPA concentrations. The Nasreddine formula [3] was superior to the Parent formula [16] in the present study, predicting free VPA concentration (Fig. 4). Notably, above 15 µg/mL, the novel formula overestimated the measured values.

To the researchers’ knowledge, this was the first study to investigate the accuracy of the Nasreddine formula [3]. The Nasreddine formula was, however, less accurate compared to the formula derived from the present study to predict the free VPA, where the difference ranged from − 4.1 to 12.3, with a median of 8.4 µg/mL and an IQR of 5.3–12.2 µg/mL. The agreement between the categorization according to the present study formula for estimating free VPA and estimated VPA was moderate (κ = 0.64; 95% CI 0.49; 0.78). The novel formula demonstrated a reasonable discordance rate of 21.4% (*n* = 18/84), with 50% of cases being underpredicted. This performance was superior to that of the Nasreddine formula, which showed a discordance rate of 33.3% (28 out of 84), with 92.9% (26 out of 28) of cases being underpredicted. Notably, the Nasreddine formula [3] does not account for albumin concentration, chronic conditions, and co-medication. Participants with chronic conditions relating to lower albumin concentration and co-medication alternating VPA concentration were included in the present study-derived formula. This could explain the more accurate prediction of the free VPA by the formula devised from the present study.

As far as the researchers could ascertain, this was also the first study to investigate the accuracy of the Doré formula [6], which was less accurate in predicting the free VPA concentration when compared to the formula devised from the present study. For the study-devised formula, the discordance was notably lower at 21.4% compared to 41.7% found by the Doré formula [6]. The variable of age was not accounted for in the Doré formula. Although age was investigated in their model, they did not find this variable to improve the accuracy of their free VPA predictions. This finding differed significantly from the present study where age showed a substantial and meaningful positive association with the free fraction. Age was therefore incorporated into the formula for the present study. This could possibly explain why the present formula performed better in predicting free VPA concentrations than the Doré formula. The median age of the present study population was significantly higher at 48 years compared to the median age of 26 years in the study by Doré et al. [6], which could further explain the difference in results.

In a more recent study by Fisch et al. [20], 506 measurements for total and free VPA concentrations of patients presenting with *status epilepticus* were analyzed at a Swiss academic medical center. They found the association between the measured and estimated free VPA to be linear. However, they did report a mismatch of 39.8% when evaluating the agreement on the RRR. In this study, the predicted/estimated free VPA overestimated the measured free VPA in 30.4% of the cases. Their multivariate regression model indicated that both total VPA and albumin concentration independently affect the accuracy in predicting free VPA [20].

The novel study-devised formula may shed some light on further studies to enhance the accuracy of equation-based formulae. However, until such time, for resource-limited settings, estimating free VPA may remain the sole viable option. Incorporating age and albumin variables could potentially enhance the accuracy of free VPA prediction.

### Limitations

The study mainly included outpatients and did not evaluate acutely ill patients, such as those admitted to ICUs, with different pharmacokinetics, trauma patients, fragile patients, including pediatric cases, and patients with renal disease on dialysis and liver impairment. However, the results are not expected to differ in principle and may likely be exacerbated in these noted circumstances, especially pertaining to conditions promoting hypoalbuminemia.

The daily prescribed VPA dose remained non-significant compared to all the associations, although non-compliance could have contributed to some extent. The study did not consider the dose-concentration relationship, but rather the concentration-response relationship. However, individualized doses in milligrams per kilogram and the cumulative dose over time in a controlled prospective study may provide stronger correlations.

### Strengths

At this stage, the study has been the largest cross-sectional observational study in an adult population on chronic VPA treatment to evaluate the different VPA measurements and various calculated methods. Multi-racial participants, patients on co-medication, and those with chronic conditions were not excluded. A novel calculated method emerged from the study.

## Conclusion

These findings emphasize the importance of individualized treatment principles. Effective management requires a holistic approach. This approach integrates assessments of both total and free VPA concentrations, alongside clinical symptoms and patient-specific factors allowing for tailored dosing adjustments guiding dosing requirements that optimize therapeutic efficacy and safety.

Therefore, there is a critical need to measure free VPA concentrations whenever feasible. Alternatively, establishing reference ranges specific to the South African population would be beneficial. This approach aims to enhance clinical practice by ensuring that therapeutic decisions are based on comprehensive and accurate information, ultimately optimizing therapeutic outcomes for patients.

Equation-based methods may be the only option for resource-constrained settings to determine free VPA. Incorporating age and albumin variables could enhance the precision of free VPA prediction.

## Data Availability

Data are available from the corresponding author on reasonable request.
